# Feasibility of a Hybrid Brain-Computer Interface for Advanced Functional Electrical Therapy

**DOI:** 10.1155/2014/797128

**Published:** 2014-01-27

**Authors:** Andrej M. Savić, Nebojša M. Malešević, Mirjana B. Popović

**Affiliations:** ^1^School of Electrical Engineering, University of Belgrade, 11120 Belgrade, Serbia; ^2^Tecnalia Serbia Ltd., 11120 Belgrade, Serbia

## Abstract

We present a feasibility study of a novel hybrid brain-computer interface (BCI) system for advanced functional electrical therapy (FET) of grasp. FET procedure is improved with both automated stimulation pattern selection and stimulation triggering. The proposed hybrid BCI comprises the two BCI control signals: steady-state visual evoked potentials (SSVEP) and event-related desynchronization (ERD). The sequence of the two stages, SSVEP-BCI and ERD-BCI, runs in a closed-loop architecture. The first stage, SSVEP-BCI, acts as a selector of electrical stimulation pattern that corresponds to one of the three basic types of grasp: palmar, lateral, or precision. In the second stage, ERD-BCI operates as a brain switch which activates the stimulation pattern selected in the previous stage. The system was tested in 6 healthy subjects who were all able to control the device with accuracy in a range of 0.64–0.96. The results provided the reference data needed for the planned clinical study. This novel BCI may promote further restoration of the impaired motor function by closing the loop between the “will to move” and contingent temporally synchronized sensory feedback.

## 1. Introduction

Depending on the objective of brain-computer interface (BCI) technology used in neurorehabilitation, the two main approaches are currently accepted and BCIs are accordingly divided into the groups of assistive and restorative [1]. Assistive BCI systems can provide humans with motor impairments, an additional channel for communication, or control of their environment [2]. Such BCIs can therefore be used for a long-term substitution of the impaired motor function [3]. Examples are BCI control of functional electrical stimulation (FES) [4, 5] or orthotic devices [6], EEG wheelchair control [7–9], or BCI spelling devices [10–13]. Restorative BCIs on the other hand aim not just at “replacement” but further enhancement of the lost/impaired motor function through neurofeedback-based training of the user [1, 14, 15]. This could be achieved if user's “will to move” (i.e., movement imagery (MI) or attempt), identified from the ongoing neural activity, directly triggers contingent sensory feedback [16, 17]. It is hypothesized that this approach induces activity dependant neuroplasticity and facilitates more natural motor control through Hebbian learning principles [18–20]. Specifically, will to move extracted from the signals collected from the brain area with a lesion can be translated into the real matching-movement of the affected limb by using FES, orthosis, or a robot [21]. By mimicking natural activation patterns between the efferent cortical activity of the areas that once controlled the affected limb and precisely temporally synchronized sensory input response that activates associated afferents might lead to rewiring or strengthening of the motor networks [1, 22–24]. Combining the BCI principles and methods with conventional assistive therapeutic modalities can result in increased efficacy of such treatments [25–27]. The term hybrid BCI is used for combining different BCI control signals, or brain- and other biosignals, in order to increase reliability or number of commands available [25, 26, 28]. We have developed a hybrid BCI system and we have tested its feasibility to be used in the rehabilitation of grasping. The goal was to improve the already affirmed method of training motor function after stroke called functional electrical therapy (FET) [29].

Here we present general characteristics of FET methodology, while more details may be found in [29–34]. FET refers to the combination of FES induced life-like movements and intensive exercise in people with central nervous system lesions [29]. Exercise of the hemiplegic arm in reaching and grasping early after stroke prevents the development of “no-use” patterns and compensatory mechanisms. FES potentially enables stroke survivors to grasp objects during the period when they are not able to control hand voluntarily. FET consists of repetitive executions of predesigned tasks, during multiple sessions (30 minutes every day for three consecutive weeks). These tasks include electrically assisted impaired movements that require exercise. The externally electrically generated or assisted grasp combined with the intensive voluntary exercise that requires activation of proximal muscles of hemiplegic arm too is a basis of FET for rehabilitation of grasp leading to an improvement in activities of daily living (ADL). FET for grasp rehabilitation includes tasks that require three basic grasping strategies: palmar, lateral, and precision (pinch) grasps necessary for ADL. The palmar grasp provides the opposition of the palm and the thumb and allows holding bigger and heavier objects such as cans and bottles. The lateral grasp provides opposition of the flexed index finger and the thumb and it is used to hold thinner objects such as a key and a paper. Precision grasp is used for objects like a pen or finger food. During FET therapy, the patient's task is to repetitively pick up and use an object placed in their range of motion. The objects are selected accordingly, each for every grasping strategy. FET stimulator provides various stimulation protocols that should be applied for different grasp types. FES patterns should be chosen to support the performed grasp (one pattern per grasp type). Therefore, stimulation pattern, that is, type of grasp to be electrically induced, must be selected before each task execution. Various means were applied in the past to control or regulate the FES patterns: shoulder motion, voice recognition, electromyographic signals, respiration, joystick, position transducer, and so forth [35]. The simplest and the most common way of selecting stimulation protocol is manually, by pressing the switch either by the subject or by the therapist [35].

The processes of selection and triggering of the stimulation pattern may be performed in a more natural and automated manner by using BCI methods [36]. We have included the two commonly used control signals for BCI devices, namely, steady-state visual evoked potentials (SSVEP) [37] and event-related desynchronization (ERD) [38] to improve the control of the rehabilitation procedure and its effectiveness, respectively. We have tested the feasibility of applying a novel, hybrid BCI architecture in order to improve FET method with two additional features: automated stimulation pattern selection and FES triggering. Hybrid BCI that we propose operates sequentially in two stages. Stage I is SSVEP-based selection of the appropriate stimulation pattern for intended type of grasp. Stage II is ERD-based triggering of this stimulation pattern as an outcome of MI of the intended grasp [39–43]. The SSVEP-BCI is introduced into the hybrid system to overcome the weakness of the conventional FET procedure reflected in the need for manual or other man-made selection of predefined FES patterns for various grasp types. The ERD-BCI has a dual role. Firstly, it acts as a brain-switch that is used to trigger the FES. Second and more important role is that combining MI with contingent sensory feedback, produced by FES, in accordance to Hebbian rule, is recognized as an effective tool for further enhancement of neuroplasticity as cited previously [22, 24]. This hybrid BCI was tested in 6 healthy subjects in order to test proposed methodology and provide reference data needed for the clinical study.

## 2. Methods

### 2.1. Subjects

Six right-handed healthy subjects (5 males and 1 female, mean age 25.5 ± 1.05) participated in this study after signing an informed consent approved by the local ethics committee. None of the subjects had previous experience with the actual hybrid BCI system. Four subjects had previous experience with motor imagery and two subjects participated in the experiments with SSVEP induction.

### 2.2. Instrumentation

Ag/AgCl electrodes (Ambu Neuroline 720, Ambu, Ballerup, Denmark) applied for EEG measurements were positioned according to the international 10–20 standard, on the C3, Oz, and Cz locations. Two scalp EEG channels were used: Oz referenced to Cz (SSVEP-BCI channel) and C3 referenced to Cz (ERD-BCI channel). Ground electrode was placed on the forehead. Impedances of the skin electrode junctions were maintained below 5 kΩ. Signals were amplified 20 k times and hardware band-pass filtered over the range 0.1–40 Hz, using PSYLAB EEG8 biological amplifier combined with PSYLAB SAM unit (Contact Precision Instruments, London, UK). Signals were digitized with 500 Hz sampling frequency using NI USB-6212 (National Instruments, Austin, TX, USA) card.

### 2.3. Experimental Protocol

**Figure 1:**
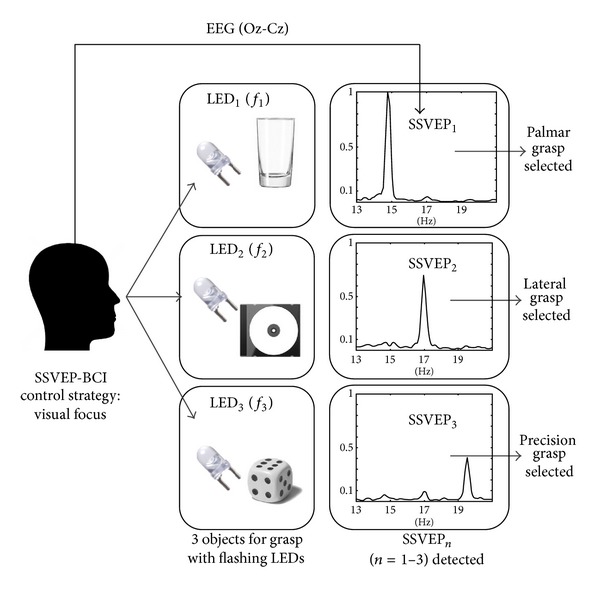
Block scheme of stage I of the hybrid BCI operation, SSVEP-BCI. While the subject gazes at one of the three objects equipped with LEDs flickering at frequencies *f*_1–3_, EEG (Oz-Cz) is analyzed for detecting associated SSVEP response. With the SSVEP detection, FES pattern for corresponding type of grasp is selected and stage II is initiated. The three graphs represent EEG (Oz-Cz) power spectra, normalized by the peak value.

**Figure 2:**
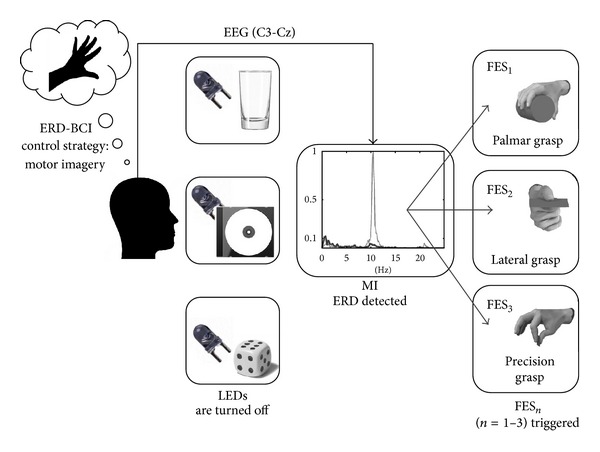
Block scheme of stage II of the hybrid BCI operation, ERD-BCI. At the beginning of stage II, all three LEDs are turned off and the subject imagines the adequate grasp for the object selected in the previous stage. If ERD is detected in the EEG (C3-Cz), previously selected FES pattern is triggered. The graph represents: EEG power spectrum during rest and EEG power spectrum during MI, normalized by the peak value (thin and thick curves, respectively).

Subjects were comfortably seated at a distance of 70 cm from 3 objects positioned on a table in a row. Objects selected for the tests, glass cup, CD case, and a playing cube required three different types of grasp: palmar, lateral, and precision, respectively. One flickering LED of a steady frequency (*f*_*n*_, *n* = 1–3) was placed beside each of the objects. Frequencies of the light flashes were *f*_1_ = 15, *f*_2_ = 17, and *f*_3_ = 19 Hz for five subjects, while for the remaining one *f*_1_, *f*_2_, and *f*_4_ were 17, 19, and 21 Hz, respectively. Before the tests, subjects were informed about the experimental procedure. At the start of each test, subjects were asked to look ahead, with all three LEDs flickering in their visual field, but without gazing at any in particular. Subjects then had to voluntarily choose an object they wanted to grasp and consequently shift their gaze toward the LED assigned to that object. While focusing on one object/LED, subjects were instructed to simultaneously reach with their right hand the object and prepare for the imagery of adequate grasping. The timings of subjects' focuses and reaches for the desired objects were manually stamped by the operator for later offline data segmentation on single trials. Immediately after the SSVEP were detected, the control command was sent to switch off all three LEDs. That was the cue for the BCI user, to start imagining the grasping of the selected object. MI tasks were imaginary palmar, lateral, and precision grasp, depending on the previously selected object. After the cue, subjects had 5 seconds to perform the MI task. If, as a result of MI, the ERD of the mu rhythm was detected, control command to trigger previously selected FES pattern was released. FES pattern activated for the desired grasp was predetermined according to the detected SSVEP (e.g., FES_1_ for palmar grasp as a result of SSVEP_1_/*f*_1_). In the situations when subjects felt that FES was activated before his/her MI, or if FES started after MI but did not feel well time-synchronized with the imagery, subjects were instructed to state “false” while being stimulated. These trials were time-stamped as FP_erd_, explained in more detail in Section 2.8. Refractory period of 3 seconds was introduced after each FES activation, during which both ERD and SSVEP BCIs were disabled. After the refractory period, LEDs were switched back on to start a new trial. Stage I of the hybrid BCI, that is, the control of the SSVEP-BCI, starts from the moment the LEDs are switched on and ends with the SSVEP detection. Stage II, control of ERD-BCI, starts when the LEDs are switched off and ends when the LEDs are turned on again. Block schemes of stages I and II are given in Figures 1 and 2, respectively.

The tasks of visual focuses were performed in subjects' own pace and order enabling the SSVEP-BCI to operate in an asynchronous mode. During the trials, subjects were instructed to choose themselves any of the three objects for grasp whenever the LEDs were on. Subjects could take a rest between the trials and proceed when they felt ready. In the situation when the SSVEP were falsely detected during the resting period, subjects were instructed to react by saying the word “false.” This was followed by manually switching the LEDs back on and time-stamping the false activation in rest as FP_*r*_ by the operator (see more in Section 2.8). In an instance that the MI of grasp was not detected within 5-second time window after LEDs' turning off, the new cycle is initiated, LEDs are switched back on, while FES is not delivered. The two-stage closed loop was repeated between 25 and 43 times per subject. During the tests, operator was notifying the trials and ensured that each subject performs at least 7 successful grasps per object. Time instances when LEDs were turned off and on were recorded for later offline analysis.

### 2.4. Electrical Stimulation System

For the electrical stimulation, we utilized INTFES multipad electrode system [44–46]. It comprises multipad electrodes and stimulator which controls both electrode pad activation and corresponding stimulation parameters. The INTFES stimulator has a Bluetooth module for high level control from PC. This feature enables us to initiate specific grasp sequence from PC application when the certain condition (command) is detected. For the task of producing different grasp types, we selected 2 square multipad electrodes, comprising 16 pads each in 4 × 4 configurations. Multipad electrodes, which acted as cathodes, were placed on dorsal and volar sides of forearm, approximately at the middle point between elbow and wrist. The common electrode (self-adhesive Pals Platinum oval electrode, 4 × 6.4 cm) was placed near the wrist. Stimulator output stage is producing biphasic charge compensated, constant current pulses. In this study, we defined pulse width of 250 *μ*s and frequency of 40 Hz, while pulse amplitudes were defined for each subject in optimization protocol. For the stimulation, we used optimal electrode configurations (active pads and current pulse parameters) for three types of grasp defined in electrode calibration protocol which precedes methodology described in this paper. During calibration protocol, automated algorithm is sequentially activating electrode pads with short train of stimulation pulses while acquiring data from inertial sensors positioned on fingers and wrist. Based on twitch amplitudes of individual joints (fingers and wrist), we optimized the stimulation pattern in order to produce the desired hand movement. The result of the calibration protocol are three configurations of pads and theirs corresponding stimulation parameters stored in PC application. Personally optimized current amplitudes were in the range 10–20 mA. The temporal parameters (time delays of initiating grasping sequences for hand opening/closing, thumb opening/closing, and wrist flexion/extension) for different grasps are predefined in accordance with natural-like movement of hand.

### 2.5. LED Controller

For the SSVEP evocation, we designed controller that communicates between PC and LEDs. The controller (PIC 18F4520) produces frequencies in a specific range (15–24 Hz) for the pulsating diodes. Flickering frequencies can be set from PC application in order to get best SSVEP response. PC application turns off all diodes when SSVEP are detected from the subjects' EEG.

### 2.6. SSVEP-BCI Operation

The SSVEP were measured with one bipolar channel Oz-Cz while the subject was gazing at a particular LED. The SSVEP responses to each LED frequency were estimated using a custom made NI LabView 2010 (National Instruments, Austin, TX, USA) based software for the EEG acquisition and online processing. The following processing steps were applied in order to detect SSVEP online. EEG channel was filtered with six 4th order Butterworth bandpass filters, with 1 Hz bandwidth. Center frequencies for the three filters were the frequencies of LED stimuli (*f*_*n*_, *n* = 1–3), while for the additional three filters, these frequencies were the corresponding first harmonics (2*f*_*n*_, *n* = 1–3). The sum of the signal amplitude filtered around the LED frequency and its first harmonic was squared and averaged in the time window of 1 s every 50 ms. Therefore, the three band-power time courses (BP_*n*_, *n* = 1–3) were extracted. Appearance of each SSVEP (SSVEP_*n*_, *n* = 1–3) results in an increase of the corresponding BP_*n*_. The SSVEP_*n*_ were detected as a BP_*n*_ raised and remained above the threshold in 10 consecutive windows, that is, for 500 ms (dwell time for SSVEP detection—DT_*s*_). Threshold values for each BP_*n*_ (TH_*n*_, *n* = 1–3) were determined manually for each subject during the 5-minute SSVEP-BCI training session prior to the tests. Additional constraint (REQ1) for accepting the SSVEP detection was that only one band-power signal (i.e., BP_1_) dwells for DT_*s*_ above the corresponding threshold (TH_1_), while the other two (BP_2_ and BP_3_) remain below their thresholds (TH_2_, TH_3_).

### 2.7. ERD-BCI Operation

**Figure 3:**
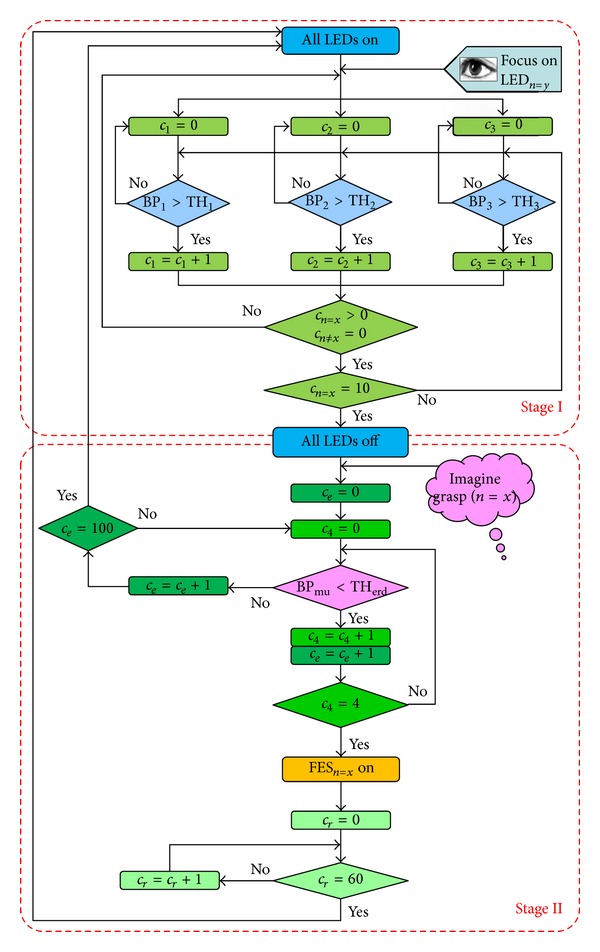
The workflow of the two-stage hybrid BCI operation. LED_n_ (*n* = 1–3) stands for LED beside the cup, CD case, and playing cube, respectively. LED_*n*=*y*_ is chosen by the subject for visual focusing. If *x* = *y* at the beginning of stage II (all LEDs off), then the correct SSVEP was detected. FES_*n*_ are the FES patterns for induction of palmar, lateral, and precision grasp, respectively. FES_*n*=*x*_ is the stimulation pattern adequate for grasping of the object beside LED_*n*=*x*_. The counters *c*_1_, *c*_2_, *c*_3_, *c*_4_, *c*_*e*_, and *c*_*r*_ compute the number of consecutive windows (iterations). The *c*_1_, *c*_2_, and *c*_3_ measure the DT_*s*_ for BP_1_, BP_2_, and BP_3_, respectively. The *c*_4_ measures the DT_erd_. The *c*_*e*_ counts the duration of 5-second interval after the LEDs are off when the subject can activate FES. The *c*_*r*_ measures the 3-second refractory period after FES_*n*=*x*_ trigger.

ERD during motor-imagery (MI) was measured using bipolar channel C3-Cz. During the 10–15-minute MI training session frequency range of the mu rhythm was determined for each subject prior to the tests. Mu rhythm was extracted using 4th order Butterworth band-pass filter. Band-power of the mu rhythm (BP_mu_) was calculated online in the time windows of 1 second every 50 ms. BP_mu_ was consequently presented as a sliding bar on the computer screen, giving subjects the continuous visual feedback on their MI task execution. Subjects were told to imagine the grasp of their right hand until power bar drops. Imageries of palmar, lateral, and precision grasp were trained. During the training session, the threshold for mu ERD (TH_erd_) was manually determined for each subject. ERD as a result of MI was detected when the BP_mu_ remained below the threshold in 4 consecutive windows, that is, for 200 ms (dwell time for ERD—DT_erd_). The flowchart presenting the hybrid BCI operation with both sequential stages, the SSVEP-BCI and the ERD-BCI, is given in Figure 3.

### 2.8. Performance Measures of the Hybrid BCI Operation

The performance of the hybrid BCI system was evaluated with the following measures: number of true activations of the SSVEP-BCI (TP_ssvep_), number of true activations of the ERD-BCI (TP_erd_), number of false activations of the SSVEP-BCI (FP_ssvep_), number of false activations of the ERD-BCI (FP_erd_), number of false negatives for the ERD-BCI (FN_erd_), number of false activations during the resting period between the trials (FP_*r*_), and number of the successful trials (TP). TP_ssvep_ is the detection of the correct SSVEP response. If the subject reached for the object while focusing on the LED beside that object, and consequently the SSVEP evoked by that LED are detected, that was considered TP_ssvep_. FP_ssvep_ is accordingly the detection of an incorrect SSVEP response. TP_erd_ is the detection of ERD after the TP_ssvep_, with an additional requirement (REQ2): ERD detection did not occur in the first 300 ms after the cue for MI. If the REQ2 was not met, and ERD was detected in the first 300 ms after TP_ssvep_, this detection is counted as FP_erd_. REQ2 is introduced for the following reason. In the situation when the combination of TP_ssvep_ and FP_erd_ produced a successful grasp (correct stimulation pattern is selected and FES is triggered), the detected ERD certainly is not the result of MI, since with the DT_erd_ accounted for, mu power has dropped below the TH_erd_ before subject was able to perform MI. The trials that were time-stamped when the subjects reported that FES seemed out of synch with MI were accounted as FP_erd_, too. FN_erd_ is the lack of ERD detection within the 5 seconds after the cue for MI. The single trial is accounted as true positive (TP) if the following conditions were met:

1. SSVEP of the appropriate frequency were detected,
2. after the LED turning off, the ERD was detected within the 0.3–5-second time interval,
3. FES activation was not reported as “false” by the subjects themselves.

In other words, the single trial of selecting and grasping of an object can be accounted as successful (TP) only if the TP_ssvep_ is followed by TP_erd_, meaning that the subject successfully grasped the object he/she reached for and that MI and ERD were time-synched. FP_*r*_ is a false SSVEP detection during the resting period between the trials. These are reported by the subjects and time-stamped by the operator. The accuracy of the hybrid BCI operation was subsequently derived using the following equation:

(1) $$\begin{array}{c} \text{Acc}=\frac{\text{TP}}{\left(\text{TP}+{\text{FP}}_{\text{ssvep}}+{\text{FN}}_{\text{erd}}+{\text{FP}}_{\text{erd}}\right)}. \end{array}$$

## 3. Results

**Figure 4:**
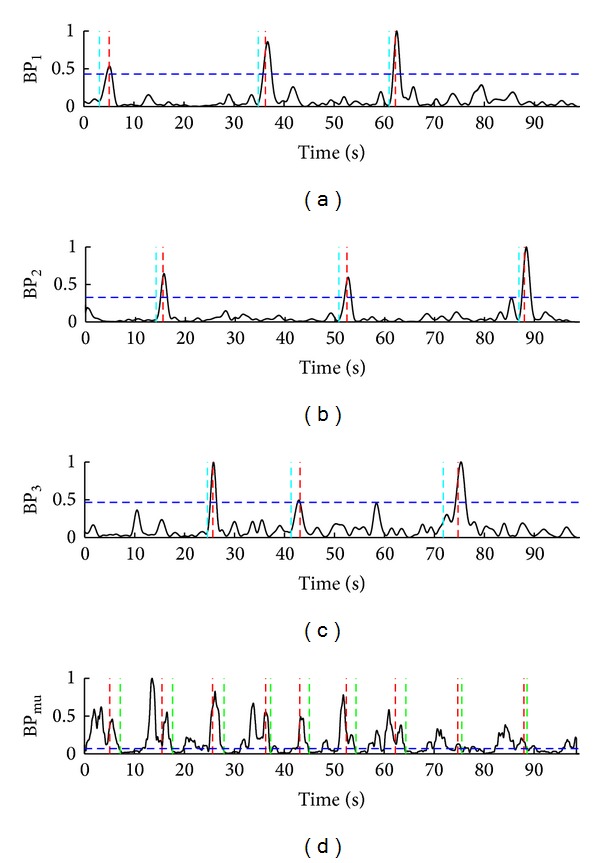
Normalized band-powers BP_1_, BP_2_, BP_3_, and BP_mu_ from (a) to (d). Nine successful trials (TP), three per each LED, for subject S2 during the representative 100-second interval are shown. Turquoise vertical dotted lines in (a), (b), and (c) point to the time instants of visual focuses on LED_1_, LED_2_, and LED_3_, respectively, while red vertical dotted lines refer to the correct detections of SSVEP_1_, SSVEP_2_, and SSVEP_3_, respectively. Red lines in (d) mark all the correct SSVEP detections (TP_ssvep_), that is, cues for MI. Blue horizontal dotted lines in (a), (b), (c), and (d) present the corresponding thresholds TH_1_, TH_2_,TH_3_, and TH_erd_, respectively. Green vertcial dotted lines mark the correct ERD detections (TP_erd_).

**Figure 5:**
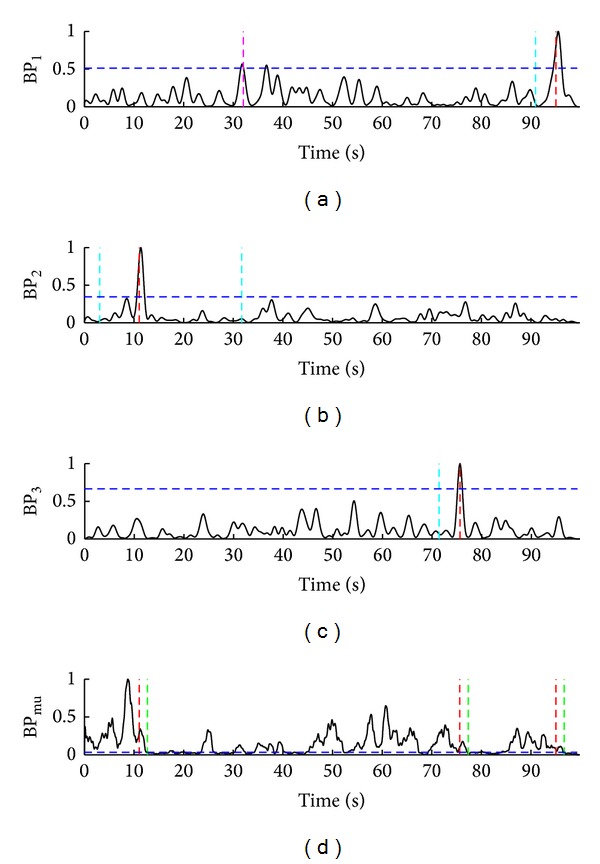
Normalized band-powers BP_1_, BP_2_, BP_3_, and BP_mu_ from (a) to (d). Three successful trials (TP), one per each LED, and one false activation of SSVEP-BCI (FP_ssvep_) for subject S5, during the representative 100-second interval are presented. (a)–(d) arrangement and color codes for turquoise, red, blue, and green are the same as in Figure 4. Pink vertical dotted line marks one incorrect SSVEP detection when subject focused and reached for the second object (LED_2_), but SSVEP_1_ were falsely detected (threshold TH_1_ was exceeded by BP_1_ for DT_*s*_).

**Figure 6:**
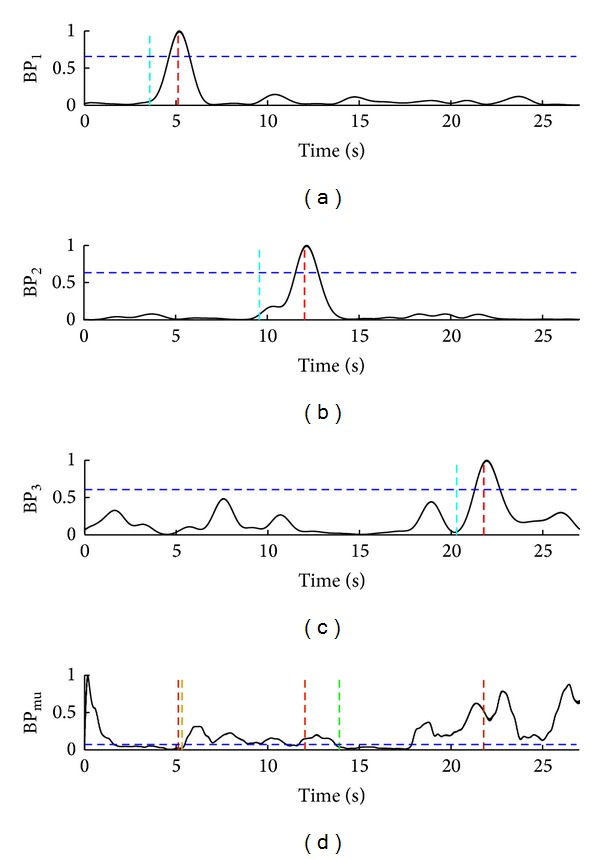
Normalized band-powers BP_1_, BP_2_, BP_3_, and BP_mu_ from (a) to (d). One successful trial (TP), one false positive ERD detection (FP_erd_), and one missed ERD detection (FN_erd_) for subject S1, during the representative 27-second interval, are presented. (a)–(d) arrangement and color codes for tirquose, red, blue, and green are the same as in Figure 4. The yellow vertical dotted line marks one FP_erd_ detection which occurred 200 ms after the cue for MI. FN_erd_ occurred after the third cue for MI; that is, ERD was not detected in the following 5 seconds after the cue for MI and FES was not activated.

The representative situations during the hybrid BCI two-stage operations are presented in Figures 4, 5, and 6. For subject S2, Figure 4 shows nine successful trials (TP) during 100-second interval. For subject S5, three successful trials (TP) and one false activation of SSVEP-BCI (FP_ssvep_) during a 100-second interval are presented in Figure 5. One successful trial (TP), one false positive ERD detection (FP_erd_), and one missed ERD detection (FN_erd_) for subject S1, during 27-second interval, are presented in Figure 6.

Table 1 summarizes the results from all measurements. Third and fourth columns include LED frequencies and number of trials (i.e., grasp attempts) per each object (LED). Frequency band used to detect ERD for each subject is in the fifth column. Total duration of the test for each subject and complete resting period between the trials are given in sixth (*T*_all_) and seventh (*T*_rest_) columns, respectively. Total resting period is a sum of all intervals in which the LEDs were on, but subject was not intentionally focusing on any of them (i.e., was not trying to select an object). Numbers of TP, FP_ssvep_, FP_erd_, FN_erd_, and FP_*r*_ per trial duration in minutes are given in eighth to twelfth columns. All subjects were able to control the hybrid BCI device with a mean accuracy of 0.78 ± 0.12.

## 4. Discussion and Conclusions

We have developed a hybrid BCI prototype to enhance the procedure of FET and tested its feasibility in healthy subjects. The system we propose is an example how to combine two BCI control signals within a hybrid architecture that provides several advantages over conventional FET. First, it enables an intuitive control of the stimulator, since the selection of an object for grasp by looking at it is a natural way to choose an item of interest. However, the main reason for introducing BCI into the conventional FET method is its potential to further enhance neuroplasticity by including motor imagery triggered FES [47]. Therefore, proposed hybrid system comprises both assistive and restorative approaches in BCI. Assistive approach is reflected in an intuitive selection of the stimulation pattern while the restorative aspect may result as the consequence of movement imagination for stimulation triggering. The fact that only 2 bipolar channels (3 electrodes) are used is favorable for practical purposes in terms of time needed to prepare the user for the treatment. While the system was designed to be used by stroke survivors mainly, the subjects in this study were healthy individuals. Evidences in several other studies support expectations that the stroke patients would be able to use and benefit from the proposed system. For example, SSVEP were recognized as robust control signals for wearable orthosis [48, 49]. Various studies showed that stroke patients are able to produce mu rhythm ERD in order to establish neurofeedback [21]. In [50] was shown that stroke patients could use mu ERD to control afferent proprioceptive feedback and that using fixed threshold for ERD detection and graded feedback depending of the detected ERD strength can lead to increase of BCI performance over the multiple session training. Authors of [51] have analyzed ERD during the cued index finger movements of the affected hand in hemiparetic patients with acute cortical and subcortical strokes, finding that the hand movements produced alpha and beta ERD in both hemispheres of all subjects over the sensory-motor areas. However, in patients with cortical lesions, for the paretic hand movements, the measured ipsilesional alpha ERD was weaker than contralesional ERD. For subcortical stroke patients no significant interhemispheric alpha ERD differences were observed. Therefore one has to take into account that depending on the lesion location, ERD strength and consequently ERD detection accuracy can be reduced in patients with cortical lesions [51, 52]. Several studies [24, 47, 53] have investigated effects of BCI hand training based on modulation of ipsilesional motor cortex mu rhythm combined with goal-directed physical therapy on motor recovery and neural reorganization in stroke patients. The results showed significant recovery of hand motor function and/or enhanced activity in ipsilesional motor cortex. The following studies have investigated combination of MI triggered proprioceptive feedback in stroke patients [24, 54, 55]. Authors of [54] were the first to report that chronic stroke patients with complete hand paralysis were able to modulate their rolandic contralateral ipsilesional mu rhythm, measured by MEG, to drive an external hand orthosis. In [55] it is reported that imagined or attempted finger movement can be translated to FES induced matching movement which resulted in motor recovery of a single chronic stroke patient. In [24] the authors used alpha sensory-motor ERD to drive hand and arm orthosis and have proven the concept that only MI temporally synchronized with the orthosis movements led to motor learning and induced plastic changes and functional improvement in chronic stroke patients. The system for electrical stimulation used in this study and the calibration protocol for generating optimal FES patterns were tested in stroke patients and the results are published in recent articles [44, 45]. Although we have successfully induced the adequate natural-like grasps in all six subjects during the tests, the generated movements were not quantitatively evaluated, as that was outside the scope of this study. However, we concluded that combination of FES and EEG recordings within the hybrid BCI is feasible since we have not observed any FES-related artifacts in the EEG frequency ranges used for the BCI control.

The proposed hybrid BCI implements asynchronous selection of stimulation pattern; that is, the user can select the object at any time and take a break if necessary without a need to switch the system off. The tests of the system were conducted with an assumption of preserved reaching. Absence of the preserved reaching can be overcome by placing the objects to be grasped in a potential users' diminished workspace. Frequencies lower than 15 Hz were not considered for use in SSVEP-BCI control because produced SSVEP could overlap with subjects' mu rhythm, used for MI detection in the second BCI stage. The REQ1 was introduced to eliminate possible false detections due to noise that induce simultaneous power rise at all three frequencies. As a consequence, false positive rate for stimulation pattern selection is lowered. The ERD-BCI operates in a synchronous (cue-based) mode given that the user has a fixed time window of 5 seconds to perform the imagery task. The ERD detections outside the window of 5 seconds after the SSVEP detection are ignored by BCI operation, allowing users to freely move between the trials without accidental FES triggering. This combination of asynchronous SSVEP BCI and synchronous ERD BCI was shown to be favorable compared to our previous work [40, 41]. Advantages of asynchronous SSVEP-BCI are its robustness, high transfer rates, and suitability for implementing BCI-based menus with multiple commands [56]. Synchronous ERD-based brain switch avoids the zero-class problem of the ERD-BCI [57]. Moreover, novel BCIs' architecture should provide potential users with more intuitive control of rehabilitation procedures and/or more independence from the assistance of the therapist [27].

## Figures and Tables

**Table 1:** Subject ID, age, LED flickering frequencies, number of trials per each LED, ERD frequency bands, *T*_all_ and *T*_rest_ in seconds, TP, FP_ssvep_, FP_erd_, FN_erd_, and FP_*r*_ per trial duration per minutes, and BCI accuracy are given in the columns. Mean (ME) and standard deviation (SD) are computed for all measurements.

1	2	3	4	5	6	7	8	9	10	11	12	13
ID	Age	LED freq. (Hz)	Number of trials	ERD freq. band (Hz)	*T*_all_ (s)	*T*_rest_ (s)	TP (min^−1^)	FP_ssvep_ (min^−1^)	FP_erd_ (min^−1^)	FN_erd_ (min^−1^)	FP_*r*_ (min^−1^)	Acc
S1	26	LED_1_: 15	7	9–13	267.71	104.25	3.59	0.45	1.12	0.45	0.22	0.64
		LED_2_: 16	8									
		LED_3_: 17	10									
S2	24	LED_1_: 15	9	9–13	271.40	101.38	5.53	0.00	0.22	0	0.22	0.96
		LED_2_: 16	9									
		LED_3_: 17	8									
S3	26	LED_1_: 15	12	8.7–13	347.37	119.09	5.53	0.35	1.38	0	0.00	0.76
		LED_2_: 16	11									
		LED_3_: 17	11									
S4	25	LED_1_: 15	14	8–13	427.95	145.15	4.49	0.00	1.54	0	0.28	0.74
		LED_2_: 16	16									
		LED_3_: 17	13									
S5	27	LED_1_: 15	9	9–13	440.13	205.17	3.14	0.14	0.27	0	0.41	0.88
		LED_2_: 16	10									
		LED_3_: 17	7									
S6	25	LED_1_: 17	14	9–13	633.67	274.19	2.84	0.66	0.47	0	0.09	0.71
		LED_2_: 18	16									
		LED_3_: 19	12									
ME	25.5		11	8.78–13	398.04	158.21	4.18	0.27	0.84	0.07	0.20	0.78
			12									
			10.50									
SD	1.05		2.83	0.4–0	136.95	68.56	1.18	0.27	0.58	0.18	0.14	0.12
			3.41									
			1.87									
